# Cycling exercise efficiency and economy: Exploring the role of phase angle

**DOI:** 10.2478/joeb-2025-0017

**Published:** 2025-12-12

**Authors:** Elie-Jacques Fares, Sarah Zaki, Jean Abi Saab

**Affiliations:** Department of Nutrition and Food Sciences, American University of Beirut, Beirut, Lebanon; Department of Medicine, University of Sherbrooke, Sherbrooke, Canada; Office of Institutional Effectiveness and Decision Support, American University of Beirut, Beirut, Lebanon

**Keywords:** Bioimpedance analysis, phase angle, delta efficiency, exercise economy, cellular health

## Abstract

**Background:**

Phase angle (PhA), derived from bioelectrical impedance analysis (BIA), is considered a non-invasive marker of cellular health and membrane integrity. Whether PhA relates to muscular efficiency during exercise remains unclear.

**Methods:**

This pilot study investigated the association between PhA and delta efficiency (DE), gross efficiency (GE), and exercise economy (EC) during submaximal cycling in 30 healthy young adults (15 females, mean age 21.4 ± 3.8 years). Whole-body and lower-body PhA were assessed using multifrequency BIA. Participants completed a graded cycling test (20–80 W) with energy expenditure determined via indirect calorimetry. DE, GE, and EC were calculated using standard procedures, and associations with PhA were examined using Pearson correlations, median-split group comparisons, and multivariable linear regression models adjusting for sex and fat-free mass.

**Results:**

Neither whole-body nor lower-body PhA was significantly correlated with DE, GE, or EC (all p > 0.05). Group comparisons based on PhA medians showed no significant differences in performance indicators. The largest observed correlation was between whole-body PhA and GE (r = −0.32, p = 0.081). Regression models adjusting for sex and fat-free mass confirmed that PhA did not independently predict DE, GE, or EC.

**Conclusions:**

PhA did not predict submaximal cycling efficiency or economy in healthy young adults. These findings suggest that cellular health, as reflected by PhA, may not directly influence muscular energetics under steady-state conditions. Future studies with larger samples and mechanistic measurements are warranted to clarify this relationship.

## Introduction

Phase angle (PhA), derived from bioelectrical impedance analysis (BIA), is recognized as a noninvasive indicator of cellular health, reflecting cell-membrane integrity and body cell mass. PhA is calculated from raw impedance measures (resistance (R) and reactance (Xc)), with higher values suggesting better cell function, hydration, and physiological integrity (1, 2). The BIA method, which employs a 50 kHz alternating current and phase-sensitive instrumentation, is well established for assessing body fluid distribution and cell mass (3).

Clinically, PhA has been associated with nutritional status, muscle function, and mortality risk, and correlates strongly with muscle mass and strength, particularly in older adults (2).

In sports science, lower-body phase angle (L-PhA) has emerged as a predictor of neuromuscular performance, especially in activities such as cycling (4, 5).

PhA also correlates positively with intracellular water (ICW) and negatively with the extracellular-to-intracellular water ratio (ECW/ICW), reinforcing its role as a marker of cellular density and fluid balance (3, 6). In healthy adults and athletes, higher PhA is associated with greater muscular strength, objectively measured physical a,ctivity levels, and higher maximal aerobic capacity, independent of body composition (7,8,9,10,11,12). These findings have led to the hypothesis that PhA may relate to muscular or metabolic efficiency during endurance exercise.

Despite these observations, the relationship between PhA and metabolic efficiency during exercise, specifically delta efficiency (DE), gross efficiency (GE), and exercise economy (EC), remains largely unexplored. Delta efficiency, defined as the ratio of mechanical work to energy expenditure during submaximal exercise, reflects muscular energy efficiency. Gross efficiency represents the ratio of work performed to the total energy expended, expressed as a percentage, while exercise economy (EC) refers to the oxygen consumption per unit of power output (13). During submaximal cycling, multiple physiological processes can influence R, Xc, and therefore PhA. Muscle contractions alter intracellular resistivity and action-potential propagation; ICW-ECW fluid shifts occur due to osmotic and pressure changes; and increases in blood flow and local muscle temperature modify tissue conductivity and membrane behavior (14,15,16,17,18,19). Under steady-state conditions these properties may stabilize, whereas more variable workloads can produce larger fluctuations in impedance values (20).

From a biophysical standpoint, Xc reflects membrane capacitance and integrity, while R reflects fluid conductivity. Favorable membrane properties reduce ATP cost for ion regulation, and optimal hydration supports oxygen and nutrient transport, mechanisms that may influence metabolic efficiency. Accordingly, PhA has been interpreted as reflecting muscle fiber quality, with higher values associated with a greater proportion of oxidative, fatigue-resistant type I fibers (21,22,23,24).

Importantly, Lukaski's dielectric model provides the formal biophysical framework linking impedance measurements to physiological states. According to Lukaski (3), R and Xc represent parallel conductive and capacitive pathways; Xc is particularly sensitive to membrane integrity and ICW content, showing disproportionate declines when membranes are disrupted or when ECW increases. Experimental and clinical data demonstrate that PhA closely tracks ICW, ECW/ICW, and biomarkers of cell proliferation, supporting its role as an integrated marker of cellular health and hydration balance (3). Through this dielectric framework, a theoretical link emerges between PhA and cycling efficiency.

However, no study has directly evaluated whether whole-body or lower-body PhA is associated with DE, GE, or EC during cycling. Therefore, this pilot study aimed to investigate relationships in healthy young adults.

## Materials and methods

### Participants

Thirty healthy adults (15 females, mean age 21.4 ± 3.8 years) participated, of whom 21 were classified as normal weight and 9 as obese. The sample had a mean height of 169.3 ± 9.3 cm, weight of 70.3 ± 19.4 kg, and BMI of 24.4 ± 6.1 kg/m^2^. Data were drawn from a larger study conducted at the Nutrition and Metabolic Laboratory at the American University of Beirut.

### Study Protocol

One week before testing, participants visited the laboratory to complete a diet, lifestyle, and medical history questionnaire and to familiarize themselves with the procedures. Measurements were standardized (fasted ~12 h, no exercise ≥24 h, no alcohol ≥24 h, post-void, at a fixed time of day (08:00 am). Height was measured using a stadiometer (Seca 213, Hamburg, Germany), body weight and composition along with whole- and lower-body phase angle were assessed by multifrequency BIA (InBody 770, InBody Co., Seoul, Korea). The InBody 770 operates at six frequencies (1, 5, 50, 250, 500, 1000 kHz). R, Xc, and PhA measurements were taken at 50 kHz frequency. 50 kHz is standard for whole-body PhA assessment as it reflects whole-body cell mass and membrane integrity. Phase angle was computed as PhA = arctan (Xc/R) × (180°/π). Participants were subsequently categorized into low- and high-PhA groups based on median cutoffs.

Reliability of our BIA protocol has previously been confirmed in our laboratory (25) for R, Xc, Z, and PA with an average coefficient of variation (CV) of 0.65%, an average intraclass correlation coefficient (ICC) of 0.99, and an average typical error (TE) of 0.7% indicating excellent reproducibility. Segmental outputs were obtained directly from the InBody analysis report: lower-limb fat-free mass (FFM_legs) was calculated as the sum of segmental lean mass from both legs, while leg fat mass percentage (FM_legs%) was determined by dividing segmental fat mass by total leg mass and multiplying by 100. Lower-body PhA was calculated as the mean of the left and right leg PhA values (Table 1).

**Table 1:** Body composition and bioelectrical characteristics of participants classified into low and high phase-angle groups. Values are presented as mean, standard deviation (SD), and range (minimum–maximum) for each parameter.

	**PhA Group (Low) n = 20**					**PhA Group (High) n = 10**				
	Mean	SD	Range	Min	Max	Mean	SD	Range	Min	Max
**Body composition parameters**										
**FFM (kg)**	48.3	10.9	32.5	33.0	65.5	54.5	13.2	34.2	39.1	73.3
**FM (%)**	27.9	11.8	39.8	7.1	46.9	25.4	11.7	34.3	8.6	42.9
**SMM (kg)**	26.7	6.6	19.6	17.6	37.2	30.6	8.0	20.5	21.4	41.9
**ECW (L)**	15.7	3.2	11.2	11.4	22.6	16.9	4.5	12.4	12.0	24.4
**ICW (L)**	21.5	6.6	22.7	13.1	35.8	24.7	8.9	24.7	14.4	39.1
**ECW/ICW**	0.8	0.1	0.2	0.6	0.9	0.7	0.1	0.2	0.6	0.8
**FFM_legs (kg)**	14.9	3.4	9.7	9.6	19.3	16.7	4.4	11.4	11.3	22.7
**FM_legs (%)**	28.1	11.4	37.9	8.6	46.5	25.4	10.5	31.1	9.7	40.8
**Bioelectrical parameters**										
**Xc/H (Ohm/m)**	38.4	6.0	20.9	29.8	50.7	40.6	7.2	25.6	29.9	55.5
**R/H (Ohm/m)**	410.3	92.5	288.3	278.2	566.5	374.9	80.0	220.4	280.6	501.0
**Whole-body PhA (degree)**	5.4	0.6	2.2	4.1	6.3	6.3	0.5	1.4	5.5	6.9
**Lower-body PhA (degree)**	6.0	0.6	2.3	4.9	7.2	6.9	0.6	1.8	5.9	7.7

### Exercise testing protocol

Participants arrived after an overnight fast, refraining from caffeine and exercise for 24 h. After 30 minutes of seated rest, energy expenditure was measured using indirect calorimetry (Cosmed CPET, Cosmed srl, Rome, Italy) with a face mask. Submaximal cycling was performed at 20, 40, 60, and 80 W (60 rpm cadence).

### Statistical Analysis

All analyses were conducted using IBM SPSS Statistics for Windows, version 30.0 (IBM Corp., Armonk, NY, USA). Statistical significance was established at p < 0.05. Participants were categorized into high (group 1) and low (group 2) phase angle (PhA) groups according to the sample median for each of the whole-body and lower-body PhA. Energy expenditure was determined using the Weir equation (26). DE was determined as the reciprocal of the slope of metabolic energy expenditure versus external work rate across cycling intensities (20–80 W), after confirming steady state and linearity (R^2^ ≥ 0.90) for each participant (27). GE was calculated as the ratio of external work rate to total metabolic energy expenditure, while EC was determined as the oxygen cost of maintaining a given submaximal workload (13). Normality was evaluated using Shapiro–Wilk tests and Q–Q plots; homoscedasticity and linearity were verified through residual diagnostics. Pearson correlations were used to examine continuous associations between PhA and efficiency outcomes. Group differences in DE, GE, and EC were tested using independent samples t-tests after confirming normality and homogeneity of variance. To adjust for confounding, separate multivariable linear regression models were constructed for DE, EC, and GE, with PhA as the primary predictor and sex and fat-free mass as covariates. Regression assumptions were checked using residual diagnostics and variance inflation factors and were adequately met.

### Informed consent

Informed consent has been obtained from all individuals included in this study.

### Ethical approval

The research related to human use has been complied with all relevant national regulations, institutional policies and in accordance with the tenets of the Helsinki Declaration, and has been approved by the authors' institutional review board or equivalent committee. The study was approved by the Institutional Review Board (BIO-2022-0200).

## Results

Pearson correlations between PhA and performance indicators were small and non-significant for both whole-body and lower-body PhA (Table 2).

**Table 2:** Pearson's correlations between whole-body and lower-body PhA and performance indicators (EC, GE, DE), including correlation coefficients (r), p-values, and sample sizes (n).

		**EC**	**GE**	**DE**
**Whole-body PhA**	r	−0.138	−0.323	0.235
	*p-value*	0.468	0.081	0.212
	*n*	30	30	30
**Lower-body PhA**	*r*	−0.149	−0.246	0.227
	*p-value*	0.432	0.190	0.228
	*n*	30	30	30

The direction of effects suggests that higher PhA tended to be associated with slightly lower EC/GE and slightly higher DE, but the precision of these estimates was limited, and none reached conventional significance. The largest observed association was between whole-body PhA and GE (r = −0.32, p = 0.081), which did not meet the threshold for statistical significance.

A strong correlation was found in our study between EC and DE (r = −0.980; p<0.001) and between whole-body PhA and Lower-body PhA (r = 0.916; p<0.001). These findings, while not the study's primary objective, confirm internal consistency among related physiological measures.

Independent samples t-tests comparing the mean EC, GE, and DE values between group 1 and group 2 for both whole-body PhA and lower-body PhA (Table 3) showed no statistically significant differences in mean values between the two groups for any of the three performance variables. Effect sizes (Cohen's d) were small for whole-body PhA comparisons and medium for lower-body PhA comparisons.

**Table 3:** Independent-samples t-tests comparing EC, GE, and DE between high vs. low PhA groups for whole-body and lower-body PhA, including means, standard deviations, 95% confidence intervals, t statistics with degrees of freedom, p-values, and Cohen's d.

**Whole-body PhA**					
	**Group 1**	**Group 2**	**95% CI**	**t–Test two–tailed**	**Cohen's d**
EC	M = 11.36 SD =1.54	M = 11.48 SD = 1.22	CI [−1.26, 1.03]	t (28) = −0.21 p>0.005	d = −0.08 small
GE	M = 12.39 SD = 1.49	M = 12.47 SD = 1.58	CI [−1.28, 1.12]	t (28) = −0.14 p>0.005	d = −0.05 small
DE	M = 24.81 SD = 2.95	M = 24.84 SD = 2.58	CI [−2.27, 2.22]	t (28) = −0.02 p>0.005	d = −0.01 small
**Lower-body PhA**					
	**Group 1**	**Group 2**	**95% CI**	**t–Test two–tailed**	**Cohen's d**
EC	M = 11.72 SD = 1.43	M = 11.04 SD = 1.37	CI [−0.38, 1.73]	t (28) = 1.31 p>0.005	d = 0.48 medium
GE	M = 12.7 SD = 1.68	M = 12.09 SD = 1.22	CI [−0.51, 1.72]	t (28) = 1.12 p>0.005	d = 0.41 medium
DE	M = 24.23 SD = 2.83	M = 25.51 SD = 2.67	CI [−3.34, 0.78]	t (28) = −1.27 p>0.005	d = −0.47 medium

Across all multivariable models (Table 4), whole-body phase angle showed no meaningful association with delta efficiency, exercise economy, or gross efficiency after adjusting for sex and FFM. For both DE and EC, neither the initial models (sex and FFM) nor the addition of PhA were statistically significant, and the change in explained variance was negligible (ΔR^2^ < 0.01, p > 0.69). For GE, sex and FFM accounted for a modest portion of the variance, driven mainly by an inverse association between FFM and GE, but adding PhA did not improve the model and its coefficient remained non-significant (p = 0.590). These findings indicate that PhA does not independently predict any of the efficiency/economy related outcomes in this sample.

**Table 4:** Multivariable linear regression models examining the association between whole-body phase angle and cycling efficiency/economy outcomes. Coefficients represent the independent contribution of each predictor to delta efficiency (DE), exercise economy (EC), and gross efficiency (GE) after adjustment for sex and fat-free mass (FFM). Unstandardized coefficients (B), standard errors (SE), standardized coefficients (β), and p-values are presented alongside the adjusted R^2^ for each model.

	**Predictor**	**B**	**SE**	**Beta**	**p**	**Adjusted R^2^**
DE	Sex	2.47	2.27	0.45	0.286	0.03
	FFM	0.15	0.1	0.64	0.141	0.03
	Whole–body PhA	0.35	1.1	0.09	0.75	0.03
EC	Sex	−1.57	1.19	−0.56	0.197	−0.01
	FFM	−0.07	0.05	−0.58	0.186	−0.01
	Whole–body PhA	−0.23	0.58	−0.12	0.691	−0.01
GE	Sex	−1.08	1.06	−0.37	0.315	0.27
	FFM	−0.12	0.05	−0.98	0.013	0.27
	Whole–body PhA	0.28	0.51	0.14	0.59	0.27

## Discussion

In this exploratory pilot study, phase angle, whether derived from whole-body or lower-body measurements, was not significantly associated with exercise economy, gross efficiency, or delta efficiency. Correlation coefficients were small and non-significant across all outcomes, and median-split comparisons between high and low PhA groups likewise showed no statistically significant differences in mean EC, GE, or DE. The largest observed association (whole-body PhA with GE, ρ = −0.32; p = 0.081) did not reach statistical significance, and effect sizes from group comparisons were small for whole-body PhA and small-to-moderate for lower-body PhA, with confidence intervals spanning zero. Consistent with these findings, the multivariable regression analyses demonstrated that PhA did not predict DE, EC, or GE after adjusting for sex and FFM. The lack of improvement in explained variance across all models further supports the conclusion that PhA contributes minimally to steady-state cycling energetics in this cohort. Taken together, these findings suggest that, within the limited range of PhA values in this cohort, PhA is not a robust predictor of steady-state cycling energetics.

These results contrast with earlier studies reporting stronger associations between PhA and physical performance in clinical or older populations. For instance, Fernández-Jiménez et al. (28) observed that lower PhA values were linked to reduced handgrip strength and a higher risk of malnutrition among hospitalized patients, while Unterberger et al. (29) reported a positive association between PhA and gait speed and chair-rise performance in older adults. Yamada et al. (1) also found that PhA correlated more closely with habitual physical activity than with isolated performance tests. The absence of similar associations in the present study may reflect the homogeneous, healthy, and physically active characteristics of our participants, whose narrow variability in PhA and physiological function likely limited the ability to detect small effects.

Our findings also challenge the theoretical expectation that higher PhA, interpreted as a proxy for cellular integrity, membrane capacitance, and favorable fluid distribution, would correspond to improved muscular efficiency during endurance exercise. While PhA has been associated with performance status, muscle quality, and training adaptations in other contexts (30,31,32), its explanatory power for EC, GE, and DE under standardized submaximal cycling appears limited in this population. One possible interpretation is that PhA reflects aspects of cellular health that do not directly constrain the metabolic-mechanical coupling captured by EC, GE, and DE at steady state. Alternatively, the link between PhA and cycling energetics may be influenced by variables such as training volume, cadence selection, muscle fiber composition, or neuromuscular coordination, factors that affect efficiency independently of global cellular properties (33).

Methodological factors may also account for the null results. The use of a median split, while convenient for interpretability, reduces statistical power and may obscure dose–response trends that would be more evident if PhA were analyzed as a continuous variable (34). Moreover, although key assumptions of normality, linearity, and homoscedasticity were verified and steady-state conditions confirmed (R^2^ ≥ 0.90), residual variability in testing conditions, such as hydration status, dietary intake, or day-to-day physiological fluctuations, could have attenuated associations between PhA and performance metrics (35).

Despite these limitations, the consistent absence of significant relationships across analyses indicates that any true association between PhA and steady-state cycling energetics is likely minimal within the tested range. Given the wide confidence intervals and the exploratory nature of the study, these findings should be regarded as hypothesis-generating rather than confirmatory.
